# Effect of Different Light Qualities on Growth, Pigment Content, Chlorophyll Fluorescence, and Antioxidant Enzyme Activity in the Red Alga* Pyropia haitanensis* (Bangiales, Rhodophyta)

**DOI:** 10.1155/2016/7383918

**Published:** 2016-08-23

**Authors:** Huanyang Wu

**Affiliations:** ^1^College of Environment and Energy, South China University of Technology, Guangzhou, Guangdong 510006, China; ^2^Key Laboratory of Pollution Control and Ecosystem Restoration in Industry Clusters, Ministry of Education, Guangzhou, Guangdong 510006, China

## Abstract

Spectral light changes evoke different morphogenetic and photosynthetic responses that can vary among different algae species. The aim of this study is to investigate the photosynthetic characteristics of the red macroalgae grown under different spectrum environments. In this study,* Pyropia haitanensis* were cultured under blue, red, and green LED and fluorescent tubes light. The growth rate, photopigment composition, chlorophyll fluorescence, and antioxidative enzymes activities in different light spectrums were investigated. The results revealed that growth rate was significantly higher in the thalli grown under blue, green, and fluorescent tubes light. Contents of Chl a and phycobiliprotein in red light were lower among all the growth conditions. Furthermore, a striking increase in SOD and CAT activity was observed in red light treatment along with the NPQ increase. The results revealed that the photosynthetic efficiency and increased growth rate of* P. haitanensis* benefitted from light spectrums such as blue, green, and fluorescent tubes light by pigment composition and photochemical efficiency manipulation, whereas red light has disadvantageous effects. Accordingly, the results for improving quality and the economic yield of algae species in some extent and the combination of different wavelengths could allow better economic resource exploitation.

## 1. Introduction

Light characteristics (spectral quality, quantity, and duration) have a profound influence on plant and seaweed metabolism and development [1, 2]. Usually, the blue-green light wavelength penetrates deepest into the water as shorter and longer wavelengths are more absorbed by water molecules or scattered by particles. Photosynthetic organs of algae have the various capabilities to adapt to these different light wavelengths.

Among various light spectra, red and blue wavelengths play an important role in the photosynthesis and photomorphogenesis, thus influencing plant development and metabolism [3]. It has been reported that the bright red and blue light can increase the ratio of fucoxanthin to Chl a and Chl c : a ratio in* Laminaria hyperborea* [4]. Furthermore, Barufi et al. [5] reported that growth rates were higher in* Gracilaria birdiae* exposed to white light and red light could be utilized as a reproductive inductor to produce tetraspores. Green light could also be beneficial for algal growth of* Halymenia floresia*. Chl a, *α*-carotene, and lutein contents were induced by white or green light and PC and PE synthesis was excited by blue light and blue or red light, respectively [6]. Red light has the potential role of stimulating lipid production of* Ettlia oleoabundans* as well [7].

Previous results suggest that red, green, and blue light have special influence on regulating algae growth and photosynthetic pigments synthesis. Although there have also been some investigations about influences of different light wavelengths on photosynthetic efficiency and chlorophyll content and growth in photosynthetic prokaryotes and eukaryotes [8–10], the effects of light spectrum in red macroalga research have been limited, especially the coupling effect between photosynthesis and antioxidant system.

This study focused on the* Pyropia haitanensis* (Bangiales, Rhodophyta), one species of marine red macroalga, which is an economically important species primarily due to being an economically important seaweed used for food and is the principal species cultivated on a commercial scale in southern China [11]. The aim of this study is to illustrate the effect of different light spectrum on chlorophyll fluorescence characteristics, the physiological and growth parameters of* P. haitanensis*, and the effect of lighting on the amount of growth rate, photopigment composition, chlorophyll fluorescence, and antioxidative enzymes activities in a different light spectrum. These results may help us realize the physiological status of the macroalgae providing data for application of natural populations as well as for the improving culture industry of this species on the coast.

## 2. Materials and Methods

### 2.1. Seaweed Materials

*Pyropia haitanensis* was gathered in Shantou, China (23°26′ N, 116°64′ E). The algae were lightly rinsed and cleared of visible epiphytes and of any accumulated sediments and then placed in a cooler containing some fresh seawater during the transportation to the laboratory (about 3 h, 4°C). The algae were preserved in a glass aquarium tank containing filtered natural seawater (salinity 3%) appended with 200 *μ*M NaNO_3_ and 20 *μ*M NaH_2_PO_4_ (final concentration) in a controlled environmental compartment. Light availability was provided by timer-controlled fluorescent tubes (100 *μ*mol photos m^−2^ s^−1^), operated on a 12 h on-and-off photoperiod. Water motion was provided by aeration and culture seawater was updated every 2 days and temperature was kept at 15 ± 1°C in the incubator. The algae were preserved for 3 days prior to the start of the experiments. Only intact and healthy thalli were selected for the subsequent experimental treatments.

### 2.2. Experimental Design

5 g fresh weight (FW) algae were transferred to a flask containing 5 L filtered seawater (three replicates per light treatment), for a starting biomass density of 1 g FW L^−1^. Different light quality control units were independently designed in growth cabinets. The cultures were irradiated at 100 *μ*mol photons m^−2^ s^−1^ for all treatment groups using LED arrays (Edlan Lighting Technology Co., China), red (R, 640~680 nm), blue (B, 420~460 nm), and green (G, 490~530 nm) light, and fluorescent tubes (CK) light. Light apparatus were affixed to a ceramic and steel support to facilitate efficient heat transfer to the mounting substrate. Each light treatment employed 3 replicates. All the cultures were preserved at 15 ± 1°C and a light/dark regime of 12/12 h, and the seawater nutrients and frequency of water changes were the same as indicated above. Seaweeds were harvested and used for experimental measurement after 7 days of cultivation.

### 2.3. Growth Rates

Biomass (fresh weight, FW) was measured at the end of incubation, and the mean relative growth rate (RGR) was assessed according to the formula RGR (% day^−1^) = ln⁡(*W*_*t*_/*W*_0_)/*t* × 100, where *W*_0_ refers to the initial FW and *W*_*t*_ to the FW after *t* days.

### 2.4. Chlorophyll Fluorescence Measurements

Detection of chlorophyll fluorescence was made using a pulse modulation fluorometer (JUNIOR-PAM, Walz, Germany). At least four algal samples were used for each measurement of chlorophyll fluorescence, and the algae were acclimated to dark for 10 min before being detected. The maximum quantum yield of photosystem (PS) II of* P. haitanensis* was estimated as *F*_*v*_/*F*_*m*_ and photochemical quenching coefficient (qP) and nonphotochemical quenching coefficient (NPQ) were also determined [12]. The rapid light curves (RLCs) consisted of the fluorescence response to eight different and increasing actinic irradiance levels over the range of 0~820 *μ*mol photons m^−2^ s^−1^. The parameters of the RLCs were calculated following the formula from Jassby and Platt [13]: rETR (relative electron transport rate) = rETR_max_ × tan⁡*h*(*α* × *I*/rETR_max_), where rETR_max_ is the saturated maximum rETR, tan⁡*h* is the hyperbolic tangent function, *α* is the initial slope of the RLC (the efficiency of the electron transport), and *I* is the incident irradiance.

### 2.5. Pigment Estimation

For chlorophyll a (Chl a) and carotenoid (Car), about 0.1 g (FW) of thalli was ground in 5 mL 95% ethanol and extracted at 4°C in darkness for 24 h. For phycoerythrin (PE) and phycocyanin (PC), 0.1 g (FW) of samples was ground in 10 mL extraction buffers (0.1 M phosphate buffer, pH = 6.8) at 4°C. The extract was centrifuged at 5000 ×g for 10 min and then used to determine the contents of Chl a, Car, PE, and PC using an ultraviolet spectrophotometer (UV-180, Shimadzu, Japan). Chl a and Car contents were analyzed according to Wellburn [14], and PE and PC contents were analyzed according to Siegelman and Kycia [15].

### 2.6. Antioxidant Enzyme Activities

Superoxide dismutase (SOD) activity was examined by measuring the ability to inhibit reduction of nitro blue tetrazolium (NBT), following the method of Beauchamp and Fridovich [16]. One unit of enzyme activity was defined as the amount of enzyme required to inhibit the photoreduction of NBT by 50%, and the unit is U mg prot^−1^ [16]. Catalase (CAT) activity (U mg prot^−1^) was analyzed by using a ferrothiocyanate method of Cohen et al. [17] with some modifications. Protein content was analyzed by the Bradford method [18].

### 2.7. Statistical Analyses

All the data were expressed as means ± SD (*n* ≥ 3), and one-way ANOVA and Tukey tests were used to analyze differences among treatments by using SPSS 17.0. The significance level was set at *P* < 0.05.

## 3. Results

### 3.1. Growth

**Figure 1:**
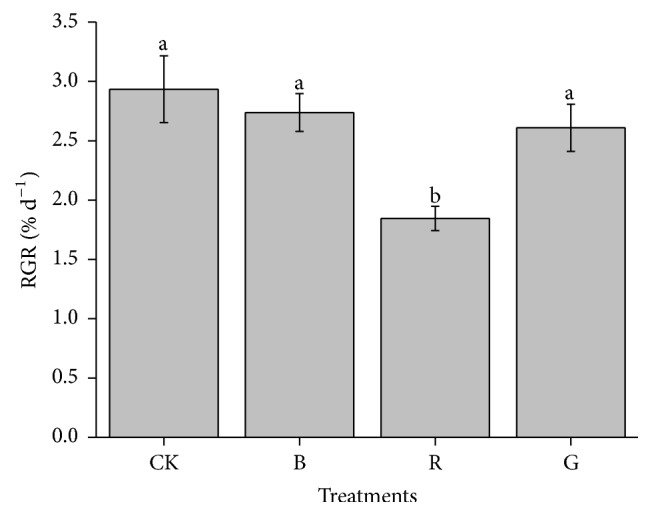
Mean relative growth rate (RGR) of* Pyropia haitanensis* as a function of the different light source (comparison via ANOVA) during laboratory culture. Values are mean (±SD), *n* = 3. Different letters indicate statistical significance (*P* < 0.05). Error bars are standard deviations.

*Pyropia haitanensis* maintained a positive growth during the period of culture for all the thalli grown under experimental conditions (Figure 1). The growth rate of the thalli grown at CK control showed higher value (2.9% d^−1^) among all experimental conditions. No significant difference was observed among CK, B, and G grown (*P* > 0.05), whereas the RGR presented the lowest value among all experimental groups when the thalli were grown at R light (*P* < 0.05).

### 3.2. Chlorophyll Fluorescence

**Figure 2:**
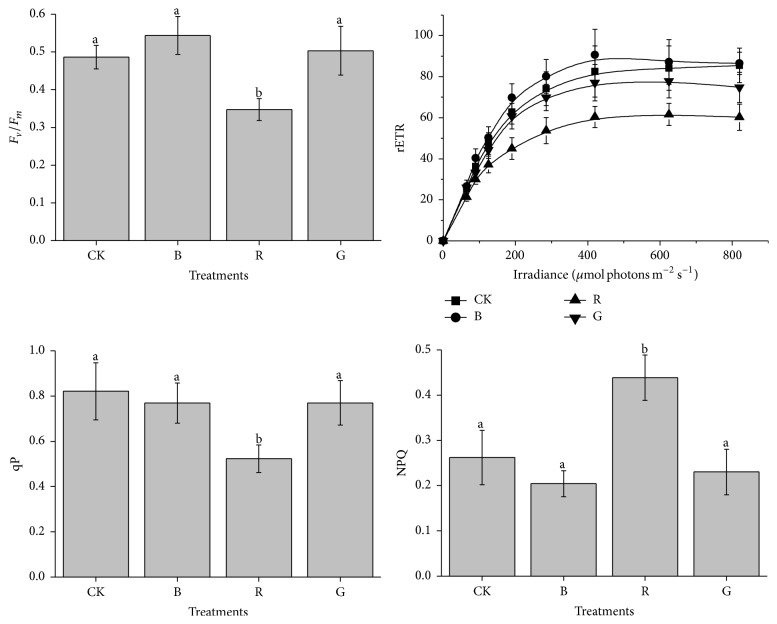
Maximal photochemical yield (*F*_*v*_/*F*_*m*_), photochemical quenching (qP), nonphotochemical quenching (NPQ), and rapid light curves (RLCs) of* Pyropia haitanensis* as a function of the light source (comparison via ANOVA) during laboratory culture. Values are mean (±SD), *n* = 9. Different letters indicate statistical significance (*P* < 0.05). Error bars are standard deviations.

Figure 2 and Table 1 illustrated the maximal photochemical yield (*F*_*v*_/*F*_*m*_), photochemical quenching (qP), nonphotochemical quenching (NPQ), and rapid light curves (RLCs) as a function of measuring photosynthetic capacity for* Pyropia haitanensis* grown at different light quality treatments. There were no significant differences among *F*_*v*_/*F*_*m*_, rETR_max_, and qP value of* P. haitanensis* in cultures maintained in CK, B, and G grown (*P* > 0.05). *F*_*v*_/*F*_*m*_, rETR_max_, and qP, however, were significantly suppressed when the thalli were grown at R light control (*P* < 0.05). Conversely, when the thalli were grown at R light control, the NPQ showed the highest value, which was statistically different as compared with the other treatments (*P* < 0.05). No significant differences of NPQ were examined among CK, B, and G grown (*P* > 0.05). Furthermore, for the parameters of RLCs thalli of* P. haitanensis* varied clearly with different light quality treatments. Compared with others, the thalli grown at CK, B, and G group had higher rETR_max_ and the initial slopes of RLCs (*α*) value and these two parameters, however, were significantly suppressed when the algae thalli were grown at R light surroundings (*P* > 0.05).

### 3.3. Pigment Experiments

**Figure 3:**
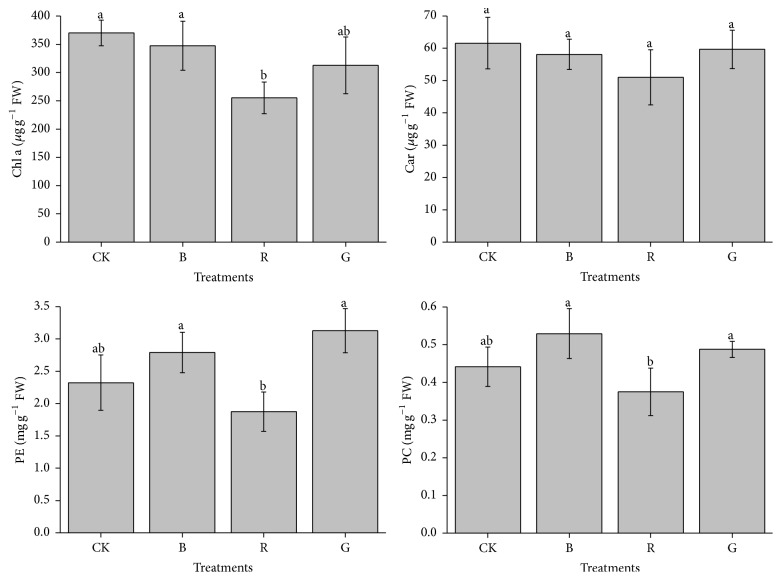
Chlorophyll a (Chl a), carotenoid (Car), phycoerythrin (PE), and phycocyanin (PC) of* Pyropia haitanensis* as a function of the light source (comparison via ANOVA) during laboratory culture. Values are mean (±SD), *n* = 3. Different letters indicate statistical significance (*P* < 0.05). Error bars are standard deviations.

Pigment contents of* Pyropia haitanensis* for different experimental condition groups are summarized in Figure 3. Higher Chl a contents were observed in thalli grown at CK and B control, whereas the Chl a contents decreased when the algae thalli were grown at R light (*P* < 0.05). For the G control, no significant differences of Chl a contents were investigated compared with others. The results of Car were not the same as Chl a. There were no significant differences when the algae thalli were grown in any light conditions (*P* > 0.05).

The PE levels in G light group possessed significant average values with 45% higher than R light treatments; significant difference values were observed between G and R light treatments (*P* < 0.05). Moreover, slight decreases of PE levels were discovered in B light treatments compared to G light group, but no significant differences were detected in these two treatments (*P* > 0.05). Likewise, for PC contents, higher mean value was observed when the algae thalli were grown under B and G light. However, slightly higher PC levels were discovered in B light treatments compared to the G light group, which is dissimilar with the PE result.

### 3.4. Antioxidant Enzyme Activity

**Figure 4:**
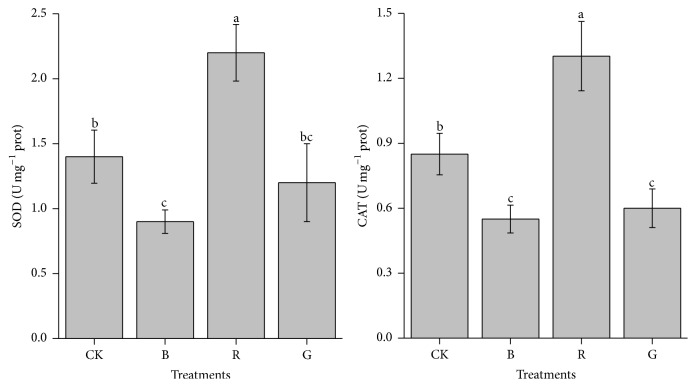
Superoxide dismutase (SOD) and catalase (CAT) activity of* Pyropia haitanensis* as a function of the light source (comparison via ANOVA) during laboratory culture. Values are mean (±SD), *n* = 3. Different letters indicate statistical significance (*P* < 0.05). Error bars are standard deviations.

The change in antioxidant enzyme activity was different according to the different light quality cultivations (Figure 4). The SOD and CAT experimental results are more similar, comparatively. For the thalli grown at R light, the SOD and CAT activity was extremely high compared with other experimental treatments (*P* < 0.05). Additionally, the maximum values of CAT activity in R light grown treatments were beyond two times that of B and G grown algae, and the effects of light quality on SOD activity of* P. haitanensis* thalli grown followed a similar manner as that of CAT, whereas no significant differences of SOD and CAT activity were observed in B and G light cultivation (*P* > 0.05), although these two cultivation groups presented a lower value of SOD and CAT activity.

## 4. Discussion

The present results suggest that thalli of* Pyropia haitanensis* are able to adjust their photosynthetic presentation to accommodate various light spectra, and the results revealed that CK, B, and G lighting was more efficient in promoting the algae growth than the thalli grown under R lighting. Similarly, some studies have shown that B and G light could play an advantageous role on red algae growth and their development [19]. Moreover, Pearson et al. [20] reported that* Silvetia compressa* releases gametes during B or in low R/B light ratios exposure, showing significant influences of light wavelength on algal growth. G light resulted in the highest algal growth rates of* Halymenia floresia* [6]. It has also been reported that monochromatic B LED-light could produce the highest biomass and RGR of* Botryococcus braunii *[21]. Additionally, the enzymes activities involved in carbohydrate metabolism of microalgae could be regulated by B light [22], thus most likely influencing the growth of seaweeds.

It has been shown that most green algae grow in the epilittoral or upper zone of water; however brown algae grow often in deeper water and many red algae can be described as subtidal algae [23, 24]. In the subtidal zone, where B and G light prevails, the specific photopigment of the red algae allows efficient absorption [25]. In addition, several red algal species growth rates and photosynthesis depend on the light quality during the culture period and on the pigment composition under these conditions [19]. Although the light requirements were very low in G and B light for all red algae analyzed, the action spectrum of growth followed the photosynthetic action spectrum, with maximum efficiencies in G and B wavebands, corresponding to the spectrum distribution occurring in deep coastal seawater [19]. Nonetheless, contrary to our results, Kim et al. [26] reported that* Gracilaria tikvahiae* presented an inferior growth rate for algae grown under B light as compared to those grown under R, G, and fluorescent light. This could be due to algae absorption characteristics depending also on several other factors, especially the thallus morphology, thickness, structure of photosynthetic system, and so forth, as it applies more to coastal waters than oceanic waters [27].

The higher growth rate of* P. haitanensis* in CK, B, and G light compared to others can be explained to some extent by the higher photochemical electron transport rate and thereby a higher efficiency of photochemical action and photosynthetic efficiency. The *F*_*v*_/*F*_*m*_ (photosynthetic efficiency), *α* (light use efficiency, the slope of the photosynthesis versus irradiance curve), rETR_max_ (defined as the photosynthetic capacity), and qP (photochemical quenching) are frequently used to represent the maximum photochemical efficiency of PSII and proportion of oxidized (open) reaction centers with PSII used as an index of photosynthetic efficiency [28].

In this experiment, *F*_*v*_/*F*_*m*_, *α*, rETR_max_, and qP were significantly decreased at R light growth, with the intense suppression in RGR, which implies that a dwindle is indicative of the decline in photosynthetic efficiency on the growth of* P. haitanensis*. Furthermore, the suppressed qP value demonstrated that R light irradiance diminished the reoxidizing *Q*_*A*_ capacity. This effect may be on account of the inhibition of Calvin cycle action, as indicated by the reduction in CO_2_ assimilation rates, which suggests R light irradiance induction pressure on PSII. This contributed to the closure of PSII reaction centers [29], thereby decreasing the probability of electron transport from photosystem II to photosystem I and beyond [30]. As researchers showed, B light could augment the number of pigment systems per electron transfer chain, whereas R light blocks Chl b synthesis and causes a decrease in the function of the light-harvesting system [31].

The NPQ is an important parameter of plant stress response, which indicates the distribution and the strength of the intrathylakoid pH gradient and the ability of chloroplasts to dissipate excess excitation energy as heat. This is similar to leaf, which is linearly associated with energy dissipation in tissues and is considered as requirement in protecting the cell from light-induced damage [32]. In our studies, significant decreases in *F*_*v*_/*F*_*m*_ and increases in NPQ occurred in R light treatment, proposing that R light caused severe abatement of photosynthesis to* P. haitanensis*, as well as an augmentation in thermal dissipation in PSII. Furthermore, this mainly suggests that* P. haitanensis* thalli grown at R light were responsive to photoinhibition and can cause the ROS generation.

Superoxide dismutase (SOD) can catalyze the dismutation of superoxide into oxygen and hydrogen peroxide, while catalase (CAT) is accountable for degrading hydrogen peroxide into water and oxygen. These two enzymes cooperate to convert ROS to H_2_O, thus enabling the algae to protect themselves against oxidative stress [33]. The influence of different spectra on the antioxidant enzymes activity in algae has also been studied with high SOD and CAT activities found in the light wavelengths range 440~480 nm for zooxanthellae [34]. In this experiment, a striking rise in SOD and CAT activity was observed in R light treatment compared with others. These results showed that the high activity of SOD and CAT is doubtless in response to the oxygen evolving photosynthetic activity in the R light. This is correlated with the NPQ increase and higher Car content, which could help to quench ROS, causing less production of ROS in the* G. lemaneiformis *[35].

Light is important for the chlorophyll synthesis, and alga pigment synthesis is regulated by various photoreceptors that absorb lighting for different wavelengths [36, 37]. In this investigation, the effects of light on the Chl a content of* P. haitanensis* presented a higher effect in CK, B, and G light treatment which was as marked as those on growth rate. López-Figueroa and Niell [37] have also shown that chlorophyll synthesis was induced by B light in red algae* Corallina elongata* Ellis et Soland and* Plocamium cartilagineum* (L.). Similarly, Kim et al. [26] proved that chlorophyll concentration was induced under G and R + B LED-light grown in* G. tikvahiae*. However, the Car content in R light treatment was not significantly decreased. It is possible to partially attribute this to the augmentation of the NPQ value. Car have a vital protective role of PSII as carotenoids can deactivate triplet chlorophyll and singlet oxygen [38].

The higher PE and PC contents were examined in B and G light grown, as expected if the red algae respond to decreased lighting intensity in the PE absorption spectrum of* Pyropia umbilicalis *[39]. Moreover, Tsekos et al. [40], Franklin et al. [41], and Barufi et al. [5] also found that B light could, respectively, increase the PE and PC concentration of* Pyropia leucosticta*,* Chondrus crispus,* and* Gracilaria birdiae*. B and G light help promote the nitrogen accumulation, and these wavelengths could induce soluble protein and phycobiliproteins formation [6]. The manifestation of the various types of photopigments in the photosynthesis complexes and their arrangement in both PSI and PSII are responsible for different photosynthetic efficiencies of various light wavelengths which affect photosynthetic activity in macroalgae.

In conclusion, R light has disadvantageous effects on the photosynthetic efficiency and increased growth rate in thalli of* P. haitanensis*. Both antioxidant enzymes activity and the NPQ coefficient were increased.* P. haitanensis* can benefit from blue, green, and fluorescent tubes light. The light wavelength treatments in mariculture of red macroalgae can be used to operate pigment composition and enzymes activity, and further investigations are required to disclose how the spectral light regulates the biosynthesis process.

## Figures and Tables

**Table 1:** Maximum relative electron transport rates (rETR_max_) and efficiency of electron transport (*α*, the initial slope of the RLCs) of *Pyropia haitanensis* grown at different light quality (comparison via ANOVA). Data are the means ± SD (*n* = 9); different letters indicate significant difference (*P* < 0.05).

Light treatments	Parameters	
	rETR_max_	*α*
CK	78.56 ± 8.31^a^	0.17 ± 0.01^a^
B	81.48 ± 9.55^a^	0.18 ± 0.02^a^
R	50.03 ± 4.87^b^	0.12 ± 0.01^b^
G	76.79 ± 8.49^a^	0.16 ± 0.02^a^
